# Long‐term benefit from adjuvant tamoxifen therapy for ER+ HER2− breast cancer by PR positivity

**DOI:** 10.1002/ijc.70409

**Published:** 2026-03-05

**Authors:** Anna E. Nordenskjöld, Magdalena Ríos‐Romero, Huma Dar, Tommy Fornander, Gizeh Perez‐Tenorio, Helena Fohlin, Olle Stål, Julia Tutzauer, Linda S. Lindström

**Affiliations:** ^1^ Institution of Clinical Sciences, Department of Oncology Sahlgrenska Academy at Gothenburg University Gothenburg Sweden; ^2^ Department of Oncology and Pathology Karolinska Institutet Stockholm Sweden; ^3^ Breast Center, Karolinska Comprehensive Cancer Center Karolinska University Hospital Stockholm Sweden; ^4^ Department of Biomedical and Clinical Sciences Linköping University Linköping Sweden; ^5^ Department of Oncology Linköping University Linköping Sweden; ^6^ Regional Cancer Center South‐East Sweden, Department of Biomedical and Clinical Sciences Linköping University Linköping Sweden

**Keywords:** breast cancer, estrogen receptor, long‐term endocrine therapy benefit, progesterone receptor, tamoxifen therapy

## Abstract

We aimed to evaluate the long‐term tamoxifen benefit by progesterone receptor (PR) levels in postmenopausal lymph node‐negative breast cancer patients with estrogen receptor (ER)‐positive/human epidermal growth factor receptor 2‐negative (HER2) tumors in the STO‐3 randomized trial. This is a secondary analysis of the STO‐3 trial including 559 postmenopausal breast cancer patients by PR levels. Patients were randomly assigned to at least 2 years of adjuvant tamoxifen therapy (40 mg once daily) versus no endocrine therapy in the Stockholm (STO)‐3 trial. Twenty‐five‐year distant recurrence‐free interval (DRFI) was assessed by Kaplan–Meier, multivariable Cox proportional hazard regression, and multivariable time‐varying analyses. Univariable Kaplan–Meier analysis showed a significant long‐term tamoxifen benefit for PR‐positive disease using a threshold of 10% or greater (DRFI, tamoxifen treated 85% vs. control 68%; *p* < .0001). Similarly, patients with high PR gene expressing tumors had a significant long‐term tamoxifen therapy benefit (DRFI, tamoxifen treated 84% vs. control 66%; log‐rank *p* < .001). In contrast, we report no significant therapy benefit for patients with PR‐negative disease (DRFI, tamoxifen treated 79% vs. control 70%; log‐rank *p* = .14) or low PR gene expression (DRFI, tamoxifen treated 82% vs. control 74%; log‐rank *p* = .17). Multivariable Cox proportional hazard regression modelling confirmed the univariable findings for PR‐positive disease (HR = 0.37; 95% CI [0.23–0.61]). Time‐varying analysis revealed a treatment benefit for PR‐positive disease up to 25 years (HR = 0.35; 95% CI [0.16–0.79]), but not for patients with PR‐negative tumors. PR‐positivity as determined by immunohistochemistry predicted long‐term benefit from adjuvant tamoxifen in lymph node‐negative postmenopausal breast cancer patients with ER+/HER2− tumors.

## Graphical abstract

What's new?

Tamoxifen is a cornerstone of endocrine therapy for estrogen receptor (ER)‐positive/human epidermal growth factor receptor 2 (HER2)‐negative breast cancer. Its long‐term benefits, however, may vary by progesterone receptor (PR) status. Here, using data from the Stockholm tamoxifen trials, the authors evaluated whether PR expression predicts 25‐year outcomes after 2 years of adjuvant tamoxifen in lymph node‐negative ER‐positive/HER2‐negative breast cancer patients. Sustained long‐term benefits from tamoxifen were observed for patients with PR‐positive tumors, while no significant benefits were associated with PR‐negative disease. The findings support PR status as a predictor of long‐term tamoxifen benefit in ER‐positive/HER2‐negative breast cancer.

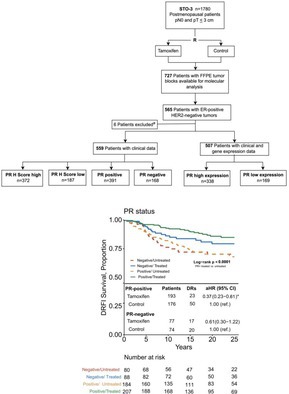

**Abbreviations**
DRFI: distant recurrence‐free interval
EBCTCG: Early Breast Cancer Trialists' Collaborative Group
ER: estrogen receptor
FFPE: formalin‐fixed paraffin‐embedded
HER2: human epidermal growth factor receptor 2
PR: progesterone receptor

## 1 INTRODUCTION

The clinically used breast cancer markers provide prognostic value for early risk, covering the first 5–10 years after primary diagnosis.^1^, ^2^, ^3^ However, it remains uncertain if the clinically used tumor characteristics can predict benefit from endocrine therapy for later risk beyond 10 years after initial primary diagnosis.^4^ As a result, there is a need to identify additional markers that can predict long‐term endocrine treatment benefit for patients with ER‐positive breast cancer.

The Early Breast Cancer Trialists' Collaborative Group (EBCTCG) reported no independent predictive value of progesterone receptor (PR) status in patients with ER‐positive tumors.^5^ However, contrasting findings from studies of adjuvant tamoxifen therapy in premenopausal patients identified PR, as assessed through immunohistochemistry (IHC), as a predictor of tamoxifen benefit.^6^, ^7^ Using tumor tissue microarrays (TMAs) from postmenopausal patients participating in the Stockholm tamoxifen (STO)‐trials we previously observed that tumors co‐expressing ER and PR were associated with a more prolonged benefit from tamoxifen compared to ER+ only tumors.^8^ Similar findings were later also suggested in whole‐tumor IHC analyses in the STO‐trials.^9^, ^10^

The predictive value of PR in assessing long‐term tamoxifen therapy benefit for ER‐positive/HER2‐negative, postmenopausal breast cancer patients remains unclear. Therefore, in this study we aimed to evaluate the long‐term benefit from tamoxifen therapy by different PR levels in the STO‐3 trial. The STO‐3 trial has 25 years of complete follow‐up and thus enables the unique assessment of long‐term benefit from tamoxifen therapy in comparison with an untreated group of patients randomized to no endocrine therapy. Patients in the STO‐3 trial have detailed and newly annotated information on the clinically used markers, as well as genome‐wide gene expression profiles.

## 2 METHODS

### 2.1 Patients

**FIGURE 1:**
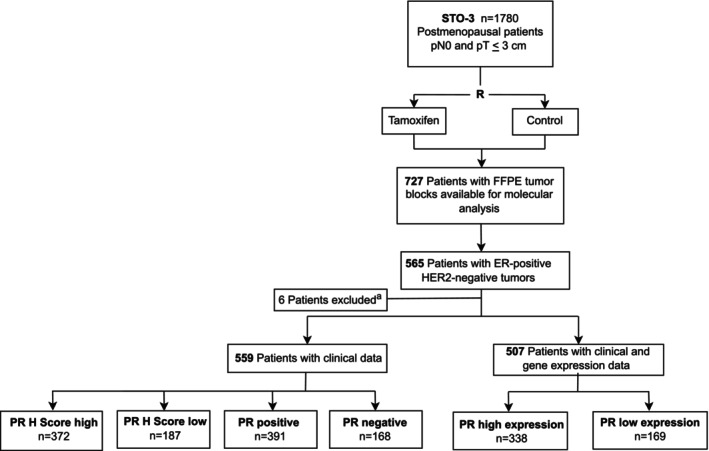
CONSORT diagram of patients from the Stockholm tamoxifen (STO) 3‐trial included in this study. FFPE, formalin‐fixed paraffin‐embedded; ER, estrogen receptor; HER2, human epidermal growth factor receptor 2; R, randomization. Superscript (a): patients with unknown PR‐status are excluded.

The STO‐3 trial (*n* = 1780) enrolled postmenopausal patients with lymph node‐negative breast cancers and tumors less than or equal to 30 mm in diameter. Patients were randomized to 2 years of adjuvant tamoxifen (40 mg daily) versus no adjuvant treatment. In 1983, patients who re‐consented and were recurrence‐free after 2 years of tamoxifen treatment were randomized to three additional years of tamoxifen or no further therapy. None of the patients received chemotherapy. Twenty‐five year follow‐up until December 31, 2016, was available for all patients from Swedish high‐quality national and regional registries of high validity and essentially complete coverage.^11^, ^12^, ^13^ The Consort diagram for the STO‐3 trial is shown in Figure 1.

Molecular analysis was possible for 727 patients in the STO‐3 trial with formalin‐fixed paraffin‐embedded (FFPE) tissue blocks from the primary breast cancer tumor (Figure 1). Patients with unknown PR status (*n* = 6) were excluded from the analyses. A total of 559 ER‐positive/HER2‐negative patients were included in this study, and 417 patients had Luminal A tumor subtype. Sub‐analyses were not conducted for patients with Luminal B subtype due to low sample size. The majority of patients (*n* = 507; 90.7%) had gene expression information available.

### 2.2 Tumor size, tumor grade, and intrinsic subtypes

Tumor size was measured as the diameter, and tumor grading was performed according to the Nottingham Histologic Score system (Elston grade).^14^ Tumors were assigned to 1 of 5 molecular subtypes (Luminal A, Luminal B, HER2‐enriched, Basal‐like, Normal‐like) using the PAM50 gene expression classification as previously described.^15^

### 2.3 Immunohistochemistry

Immunohistochemical (IHC) analysis of ER, PR, HER2, and proliferation‐marker Ki‐67 was reannotated in 2014 on whole‐tumor sections and followed standard recommended clinical protocols. The percentage of cancer cells positive for ER, PR, HER2, and Ki‐67 was scored by experienced breast cancer pathologists. According to the Swedish National Guidelines,^16^ ER‐positivity and PR‐positivity were defined by a threshold of 10% or greater, HER2‐positivity was defined as intensity 3+ by IHC, and Ki‐67 was measured in the whole stained tumor slide and categorized as low (<15%) and medium to high (≥15%). Pathologists also scored the percentage of positive breast cancer cells for each PR intensity level (0, +1, +2, or +3) compared to established standards. PR H Score was defined as the sum of the percent of PR‐positive tumor cells at each intensity level multiplied by an ordinal value corresponding to the intensity level.^17^

We also conducted supplementary analyses using the American Society of Clinical Oncology (ASCO) guidelines threshold, in which 1% or more of tumor cells staining positive on IHC is considered positive.^18^

### 2.4 PR gene expression analysis

Gene expression data was generated using custom designed Agilent arrays containing approximately 32.1 K probes, representing approximately 21.5 K unique genes from FFPE breast cancer tumor tissue. Gene expression from each chip was log2‐scaled and upper quartile normalized. In total, 652 breast cancer tumors passed the quality check. After performing Robust Multichip Average (RMA) normalization on the Agilent microarray data, multiple probes corresponding to the same gene were collapsed, selecting the probe with the highest mean expression value, as previously described.^19^ Thus, for the PR gene, which was represented by two probes, the A_23_P138938 probe was selected by using this method.

### 2.5 Statistical analysis

PR was assessed by IHC, H Score, and gene expression. The H Score and gene expression were categorized into two groups by the tertiles of the expression distribution, that is, cut‐off for the low score/expression group was the first tertile and second tertile and higher was defined as high score/expression.

The clinical endpoint was distant recurrence‐free interval (DRFI) with distant metastasis as the event of interest. The long‐term (25‐year) tamoxifen therapy benefit was assessed by univariate Kaplan–Meier (log‐rank test), multivariable Cox proportional hazard regression, and flexible parametric analysis. We report two types of *p*‐values for Kaplan–Meier analyses: (a) a global log‐rank *p*‐value across all strata and (b) pairwise log‐rank *p*‐values for the between‐group comparisons. Multivariable analyses were adjusted for standard clinical patient and tumor characteristics: age, randomization period, surgery type (mastectomy or breast conserving surgery), Ki‐67 status, tumor grade, and tumor size.

Multivariable time‐varying analysis was conducted to evaluate how risk and treatment benefit varied over the 25‐year follow‐up using flexible parametric survival modeling.^20^ The baseline log‐cumulative hazard was modelled using a natural cubic spline function of log time with 2 degrees of freedom. The treatment effect was modelled as a time‐varying coefficient with 1 degree of freedom. Model selection was based on both the Akaike Information Criterion (AIC) and the Bayesian Information Criterion (BIC). The R survival and R survminer packages were used for the Kaplan–Meier and Cox proportional hazard regression analyses, and the stpm2 R package for the flexible parametric survival modelling. Analyses were performed in R version 4.3.1. A *p*‐value less than .05 was considered statistically significant and all statistical tests were two‐sided.

## 3 RESULTS

A total of 559 patients with ER‐positive/HER2‐negative breast cancer were included and the clinicopathological characteristics are presented in Table 1. The median age was 63 years (range: 45–73), and most patients had tumor size T1 (*n* = 455, 82.3%), grade 2 (*n* = 357, 64.8%), and Ki67‐low (*n* = 423, 79.7%). The majority of patients were PR‐positive (*n* = 391; 69.9%), whereas 168 (30.1%) patients were PR‐negative. No significant differences in age, tumor grade, or tumor size were observed between the PR level groups.

**TABLE 1:** Patient and primary tumor characteristics related to PR IHC status in the STO 3‐trial.

		PR‐negative (*n* = 168)	PR‐positive (*n* = 391)	*p*‐value^a^	Total, *n* (%)
Patient and tumor characteristics					559 (100)
Age, *n* (%)	≤45	0 (0.0)	1 (0.3)	.989	1 (0.3)
	46–50	3 (1.8)	8 (2.0)		11 (1.9)
	51–55	20 (11.9)	44 (11.3)		64 (8.4)
	56–60	43 (25.6)	94 (24.0)		137 (24.5)
	61–65	45 (26.8)	113 (28.9)		158 (28.3)
	>65	57 (33.9)	131 (33.5)		188 (36.6)
Calendar period, *n* (%)	1976–1979	27 (16.1)	62 (15.9)	.988	89 (15.9)
	1980–1984	61 (36.3)	140 (35.8)		201 (36.0)
	1985–1990	80 (47.6)	189 (48.3)		269 (48.1)
Tumor size, *n* (%)	T1	133 (80.6)	322 (83.0)	.543	455 (82.3)
	T2–T3	32 (19.4)	66 (17.0)		98 (17.7)
	Unknown	3	3		6 (1.1)
Tumor grade (%)	1	34 (20.5)	92 (23.9)	.672	126 (22.9)
	2	108 (65.1)	249 (64.7)		357 (64.8)
	3	24 (14.4)	44 (11.4)		68 (12.3)
	Unknown	2	6		8
Ki67 status, *n* (%)	Low	119 (77.3)	304 (80.6)	.406	423 (79.7)
	Med/high	35 (22.7)	73 (19.4)		108 (20.3)
	Unknown	14	14		28
PR gene expression^b^, *n* (%)	Low expression	127 (87.0)	42 (11.6)	<.001	169 (27.9)
	High expression	19 (13.0)	319 (88.4)		338 (72.1)
PR H Score, *n* (%)	Low score	168 (100.0)	19 (4.9)	<.001	187 (27.1)
	High score	0 (0.0)	372 (95.1)		372 (72.9)

Abbreviation: PR, progesterone receptor.

^a^ *p*‐value calculated by Fisher's exact test.

^b^ Derived from the subset of patients (*n* = 507) with gene expression data available.

### 3.1 25‐year tamoxifen benefit by PR IHC status

**FIGURE 2:**
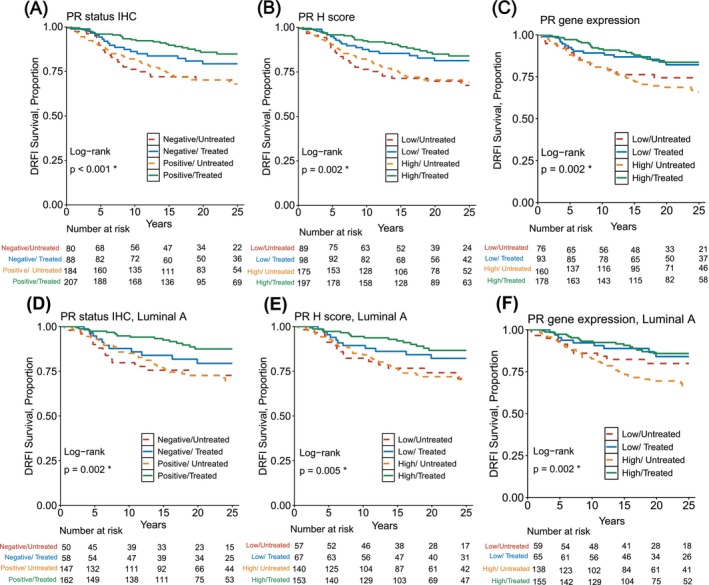
Kaplan–Meier analyses of 25‐years distant recurrence‐free interval (DRFI) in ER‐positive HER2‐negative patients randomly assigned to at least 2 years of tamoxifen therapy compared with no adjuvant endocrine therapy (control). (A) PR status by IHC including all patients, (B) PR status by IHC including patients with Luminal A tumors (C) PR by H Score including all patients, (D) PR by H Score including patients with Luminal A tumors, (E) PR levels by gene expression including all patients, and (F) PR levels by gene expression including patients with Luminal A tumors. The *p*‐values shown refer to the global comparison between all four groups.

The benefit of tamoxifen was first evaluated in groups of patients with tumors of different PR status according to IHC (Figure 2A). Tamoxifen‐treated patients with PR‐positive tumors had significantly prolonged DRFI survival (log‐rank *p* < .0001; Figure S1A) as compared to control, where PR‐positive patients' survival proportions at 25 years by DRFI were 85% and 68% for patients randomly assigned to tamoxifen or control, respectively. In the PR‐negative group, tamoxifen did not significantly prolong DRFI in Kaplan–Meier analysis (log‐rank *p* = .14; Figure S1B), with survival proportions at 25 years by DRFI at 79% and 70% for patients randomly assigned to tamoxifen or control, respectively.

We additionally conducted supplementary analyses using the ASCO threshold with 1% positive tumor cells or more defined as PR‐positive (Figure S2A). Of note, the number of patients with PR‐negative tumors was reduced from 168 to 114. For patients with PR‐positive tumors according to the ASCO cutoff, 25‐year DRFI was 83% versus 69% in the tamoxifen and control groups, respectively (log‐rank *p* < .001; Figure S2B). For patients with PR‐negative tumors according to the ASCO cutoff, tamoxifen also significantly improved DRFI, with 25‐year DRFI of 84% and 67% in the tamoxifen‐treated and control groups, respectively (log‐rank *p* = .043) (Figure S2C).

Next, the association between PR IHC status and tamoxifen benefit was assessed among patients with tumors classified as Luminal A (Figure 2B). Tamoxifen‐treated patients with PR‐positive Luminal A tumors showed significantly increased DRFI as compared to PR‐positive untreated controls (log‐rank *p* < .001; Figure S3A). Survival proportions at 25 years by DRFI were 87% and 70% for tamoxifen‐treated or untreated patients with PR‐positive Luminal A tumors, respectively. Tamoxifen‐treated patients with PR‐negative Luminal A tumors showed no significant difference in long‐term DRFI as compared to untreated controls (log‐rank *p* = .36; Figure S3B), with survival proportions at 25 years by DRFI at 79% and 73% for patients randomly assigned to tamoxifen or untreated, respectively.

**FIGURE 3:**
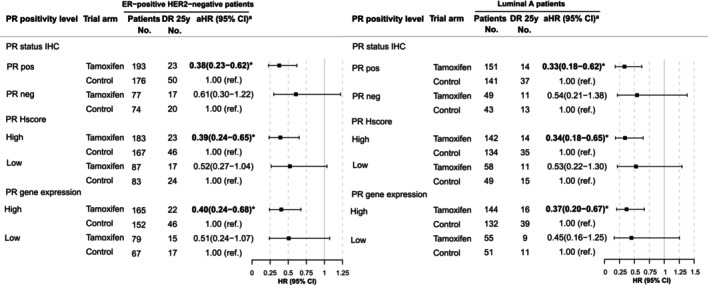
Multivariable Cox proportional hazard regression analysis of 25‐year distant recurrence‐free interval (DRFI) contrasting patients randomized to tamoxifen versus control. All ER‐positive/HER2‐negative patients and Luminal A patients only are presented. Bold and asterisks indicate significant *p* < .05. Superscript (a): adjusted for age, randomization period, tumor size, tumor grade, Ki‐67 status and type of surgery. DR, distant recurrences; aHR, adjusted hazard ratio.

Similar to the univariate Kaplan–Meier analysis, the multivariable analyses in Figure 3 show that patients with PR‐positive, as well as patients with Luminal A PR‐positive tumors had a significantly improved long‐term DRFI from tamoxifen (PR‐positive, HR = 0.37; 95% CI [0.23–0.61] and Luminal A PR‐positive, HR = 0.33; 95% CI [0.18–0.61]). No significant long‐term benefit from tamoxifen was seen for patients with PR‐negative tumors or patients with Luminal A PR‐negative tumors (PR‐negative, HR = 0.61; 95% CI [0.30–1.22] and Luminal A PR‐negative, HR = 0.54; 95% CI [0.22–1.32]; Figure 3). However, these findings must be interpreted with care due to the small number of patients available (ER+ HER2− PR‐negative patients *n* = 151; events = 37 and Luminal A PR‐negative patients *n* = 99; events = 24).

In analyses using the ASCO ≥1% threshold of PR‐positivity, multivariable models showed similar long‐term DRFI benefit from tamoxifen in PR‐positive and PR‐negative patients (Table S1).

### 3.2 25‐year tamoxifen benefit by PR H Score

Next, the treatment benefit of tamoxifen was analyzed in patients with tumors of different PR status by H Score (Figure 2C). Kaplan–Meier analysis of tamoxifen‐treated patients with high PR H Score tumors showed a significant increase in DRFI, as compared to untreated patients (log‐rank *p* < .001; Figure S1C), with survival proportions at 25 years by DRFI at 84% and 69% for patients randomly assigned to tamoxifen or untreated, respectively. Also, low PR H Score tamoxifen‐treated patients had prolonged DRFI as compared to the untreated group (log‐rank *p* = .034; Figure S1D). The survival proportions were 81% and 67% DRFI at 25 years of follow‐up for patients randomly assigned to tamoxifen or untreated, respectively.

The treatment benefit from tamoxifen in relation to PR status by H Score was then evaluated in patients with Luminal A tumors (Figure 2D). Tamoxifen‐treated patients with high PR H Score Luminal A tumors had significantly prolonged DRFI survival (log‐rank *p* < .001; Figure S3C) as compared to the corresponding controls. Survival proportions at 25 years by DRFI were 87% and 70% for tamoxifen‐treated patients or untreated, respectively. Tamoxifen‐treated patients with low PR H Score Luminal A tumors showed no significant difference in long‐term DRFI as compared to corresponding controls, with survival proportions at 25 years by DRFI at 82% and 71% for patients randomly assigned to tamoxifen or untreated, respectively (log‐rank *p* = .18; Figure S3D).

Multivariable Cox proportional hazard regression analyses of tamoxifen‐treated ER+ HER2− patients with high PR H Score tumors and Luminal A high PR H Score tumors showed significantly improved long‐term DRFI as compared to controls (HR, 0.39; 95% CI, 0.23–0.64) and (HR, 0.34; 95% CI, 0.18–0.63), whereas no significant long‐term benefit from tamoxifen was seen for patients with ER+ HER2− low PR H Score tumors (HR, 0.53; 95% CI, 0.27–1.04) or the Luminal A low PR H Score tumors (HR, 0.53; 95% CI, 0.22–1.27) (Figure 3). However, given the limited number of patients available, these results must be interpreted cautiously (ER+ HER2− low PR H Score patients *n* = 170; events = 41 and Luminal A low PR H Score patients *n* = 115; events = 26).

### 3.3 25‐year tamoxifen benefit by PR gene expression

Then, the tamoxifen benefit was determined in patients with tumors of high or low PR gene expression (Figure 2E). Tamoxifen‐treated patients with high PR‐gene expression showed a significant improvement in DRFI (log‐rank *p* < .001), with survival proportions at 25 years by DRFI at 84% and 66% for patients randomly assigned to tamoxifen and control, respectively (Figure S1E). No significant benefit from tamoxifen therapy was seen for patients with low PR gene expression, with survival proportions at 82% and 74% for tamoxifen treated versus control, respectively (log‐rank *p* = .17; Figure S1F).

The tamoxifen benefit was further analyzed in patients with tamoxifen‐treated patients with Luminal A tumors of high or low PR gene expression (Figure 2F). Patients with Luminal A tumors of high PR gene expression had significantly improved DRFI compared with control (log‐rank *p* < .001; Figure S3E). Survival proportions at 25 years by DRFI were 86% and 66% for tamoxifen‐treated patients versus untreated, respectively. In contrast, patients with low PR gene expressing Luminal A tumors did not have a significant tamoxifen therapy benefit (DRFI: 84% and 80% for tamoxifen‐treated patients versus untreated; log‐rank *p* = .43; Figure S3F).

Similar to the univariable Kaplan–Meier analyses, multivariable Cox proportional hazard regression analyses showed significantly increased long‐term DRFI from tamoxifen for ER+ HER2− high PR gene expression patients (HR, 0.40; 95% CI, 0.24–0.67) and the Luminal A high PR gene expression group (HR, 0.36; 95% CI, 0.20–0.65) (Figure 3). No significant long‐term benefit from tamoxifen was seen for ER+ HER2− patients with low PR gene expressing tumors (HR, 0.50; 95% CI, 0.24–1.06) nor Luminal A tumors with low PR gene expression (HR, 0.47; 95% CI, 0.17–1.27) (Figure 3). Also, these findings must be interpreted with care due to the small number of patients available (ER+ HER2− low PR gene expression patients *n* = 157; events = 32 and Luminal A low PR gene expression patients *n* = 115; events = 20).

### 3.4 Time‐varying multivariable analysis of tamoxifen benefit by PR positivity levels

Time‐varying analyses were performed to evaluate how the risk for distant recurrence and tamoxifen therapy benefit varies over time. Patients with PR‐positive tumors analyzed by IHC had a steady long‐term risk of distant recurrence; however, tamoxifen benefit was seen from year 5 to year 25 compared with untreated (HR, 0.35, 95% CI, 0.16–0.79 at year 25). The same trend was identified when PR was estimated within the high H Score group, in which benefit from tamoxifen was observed until the end of follow‐up (HR, 0.36, 95% CI, 0.16–0.81 at year 25). High PR gene expression levels show significant early tamoxifen treatment benefit compared with untreated (HR, 0.37, 95% CI, 0.20–0.66 at year 5). In contrast, patients with PR low H Score and low PR gene expression disease showed no significant benefit from tamoxifen early nor later in the follow‐up (see Table 2).

**TABLE 2:** Time‐varying multivariable analysis of tamoxifen therapy benefit by PR levels.

STO‐trial arm and stratification	Years	Risk of DR, aHR (95% CI)^a^
Tamoxifen vs. control in patients with PR‐positive tumors by IHC	5	0.38 (0.23–0.63)*
	15	0.36 (0.19–0.69)*
	25	0.35 (0.16–0.79)*
Tamoxifen vs. control in patients with PR‐negative tumors by IHC	5	0.59 (0.28–1.26)
	15	0.71 (0.20–2.49)
	25	0.75 (0.15–3.75)
Tamoxifen vs. control in patients with PR H Score high tumors	5	0.40 (0.24–0.66)*
	15	0.37 (0.19–0.71)*
	25	0.36 (0.16–0.81)*
Tamoxifen vs. control in patients with PR H Score low tumors	5	0.52 (0.25–1.06)
	15	0.59 (0.20–1.71)
	25	0.62 (0.15–2.52)
Tamoxifen vs. control in patients with PR gene expression high tumors	5	0.37 (0.20–0.66)*
	15	0.48 (0.23–1.002)
	25	0.54 (0.20–1.43)
Tamoxifen vs. control in patients with PR gene expression low tumors	5	0.48 (0.23–1.03)
	15	0.63 (0.19–2.10)
	25	0.68 (0.16–2.91)

*Note*: 25‐year distant recurrence‐free interval (DRFI) contrasting patients randomized to tamoxifen versus control. Bold and asterisks indicate significant *p* < .05.

Abbreviations: aHR, adjusted hazard ratio; DR, distant recurrences.

^a^ Adjusted for age, randomization year, tumor size, tumor grade, Ki‐67 status, and type of surgery.

## 4 DISCUSSION

Patients with ER‐positive and PR‐positive tumors demonstrated a marked and sustained benefit from tamoxifen therapy, with a continuous separation of survival curves between treated and untreated groups over 25 years. These findings are consistent with our previous results, where ER and PR co‐expression predicted prolonged recurrence‐free survival following adjuvant tamoxifen therapy.^8^ These results align with Bardou et al., who reported that PR‐positive was associated with improved survival in patients receiving endocrine therapy.^21^ Additionally, studies on advanced breast cancer have demonstrated higher response rates and prolonged survival in tamoxifen‐treated patients with PR‐positive tumors.^22^ Similar observations have been made in studies of adjuvant endocrine therapy, which show higher recurrence rates and shorter time to recurrence in patients with PR‐negative disease,^23^, ^24^ potentially related to increased growth factor receptor signaling in PR‐negative cases.^25^ Our data show a nonsignificant trend indicating some tamoxifen benefit also for patients with ER‐positive and PR‐negative disease. There are no clinical trials indicating specific effects of alternative endocrine therapies such as fulvestrant or aromatase inhibitors for ER‐positive and PR‐negative breast cancer, and further studies are needed to understand potential benefit.

In our study, tamoxifen therapy showed a pronounced and longstanding benefit on patients with Luminal A tumors co‐expressing ER and PR. Conversely, PR‐negative Luminal A patients appeared to experience limited benefit from adjuvant tamoxifen, although this finding should be interpreted with caution due to the small sample size. The use of the PR H Score, which accounts for staining intensity and the proportion of stained cells, did not provide additional or differential predictive value compared to the simpler metric of PR positivity defined as at least 10% stained cells. Similarly, PR gene expression analysis did not enhance predictive capacity compared to IHC‐based PR positivity. Nonetheless, the favorable outcomes observed in patients with Luminal A tumors underscore the importance of molecular characterization in breast cancer management.

Approximately 30% of postmenopausal patients in our cohort had PR‐negative disease, a figure higher than the approximately 10% reported for premenopausal disease in the Swedish tamoxifen trials.^26^ Despite this difference, PR‐positivity consistently acts as a significant predictor of tamoxifen benefit in both premenopausal and postmenopausal patients.^7^, ^8^ Engström et al. demonstrated that PR positivity—whether assessed by cytosol technique, IHC, or gene expression—predicts tamoxifen benefit in premenopausal patients.^27^

Aromatase inhibitors (AI), introduced after tamoxifen, are now widely used either alone or in sequence with tamoxifen as adjuvant therapy.^28^ Ethical considerations precluded trials comparing AIs to untreated controls. While trials comparing AIs and tamoxifen provide approximately a decade of follow‐up data, the long‐term effects of AIs remain less well understood. The BIG 1‐98 trial, with a median follow‐up of 12.6 years, reported initial benefits favoring letrozole, including reduced contralateral breast cancer incidence. However, this trend reversed beyond 10 years, potentially reflecting the long carryover effect of tamoxifen.^29^ Current evidence suggests that for a 60‐year‐old patient with PR‐positive Luminal A disease, treatment strategies such as 2 years of letrozole followed by 3 years of tamoxifen yield outcomes comparable to 5 years of letrozole monotherapy. For patients intolerant to AIs, switching to tamoxifen represents a viable alternative, leveraging its well‐documented long‐term carryover effect.^6^, ^30^

Time‐varying analyses from this study reinforce the prolonged carryover effect of tamoxifen, persisting beyond until 25 years of follow‐up in several of the groups analyzed. Tailoring adjuvant endocrine therapy to achieve optimal risk reduction is important. In this study, patients received 40 mg tamoxifen daily rather than the currently usually used 20 mg. Although the dose–response relationship for tamoxifen remains underexplored, data from the comparison of two and 5 years of tamoxifen therapy in which 20 and 40 mg daily were also compared indicate that 20 mg is sufficient for recurrence protection.^31^ Furthermore postmenopausal patients, patients with lower ER expression or PR‐negative tumors benefit from extended therapy up to 5 years, while those with high ER expression or PR‐positivity achieve similar outcomes with 2 or 5 years of treatment.^32^ For patients with ultra‐low recurrence risk, identified for example through Genomic Risk, endocrine therapy may be reduced to 2 years of tamoxifen at 20 mg daily or potentially omitted altogether.^1^, ^33^

Our findings differ from those reported in the EBCTCG overview,^5^ that may be attributed to variations in methods for assessing PR positivity, differences in patient risk profiles, age distribution, and the inclusion of adjuvant chemotherapy in several studies in addition to 5 years of tamoxifen treatment versus control in the EBCTCG trials. Further, in the overview, cytosol‐based ligand binding assays were commonly used to evaluate PR status. Notably, the EBCTCG overview reports that 21% of ER‐negative cancers were classified as PR‐positive, suggesting a high likelihood of false‐positive PR classifications associated with this technique. In contrast, immunohistochemical analysis has demonstrated that PR‐positivity in ER‐negative tumors is uncommon. Additionally, to understand the long‐term benefit of adjuvant tamoxifen therapy, follow‐up data must be both prolonged and of high quality, as exemplified by the Stockholm tamoxifen trials.^11^, ^12^, ^13^ Even if the limitations of the EBCTCG overview are considered, it is important to also note that in spite of the inclusion of a sizable number of trials, resulting in a large number of patients, the meta‐analysis did not find tamoxifen therapy predictive information from PR positivity. More studies are therefore needed to understand the role of PR and its importance on endocrine therapy benefit.

This study presents both limitations and advantages. The guidelines for treating breast cancer have changed since the patients were included in the study. Most current corresponding patients are diagnosed after mammography screening, receive breast conserving therapy, and 5 years of endocrine treatment. However, the significantly improved breast cancer outcome after adjuvant endocrine therapy, as demonstrated in the current study, is still the basis for clinical practice 50 years after the trial started. The optimal duration of endocrine therapy for patients by ER/PR levels and recurrence risks needs further study. The sample size of the ER‐positive/HER2‐negative subset from the STO‐3 trial although derived from size‐adequate trial, becomes limited in certain sub‐analyses, particularly for the Luminal A patients. Therefore, caution should be taken in results interpretation. However, the study benefits from high‐quality and long‐term patient follow‐up in the STO‐3 trial, including a control arm of patients randomized to no tamoxifen therapy. The population‐based recruitment of patients from the same geographic area in Sweden ensures suitability for comparison between the patient groups analyzed here with minimal unknown variability. This design allowed us to appropriately assess the endpoint, which was DRFI, until up to 25 years of follow‐up. Note that the patients in this study received at least 2 years of tamoxifen, whereas today's standard is at least 5 years. Our findings could be helpful for clinicians and relieving for patients that are unable to endure the total duration of endocrine therapy.

## AUTHOR CONTRIBUTIONS

**Anna E. Nordenskjöld:** Conceptualization; methodology; writing – original draft; writing – review and editing; funding acquisition. **Magdalena Ríos‐Romero:** Methodology; writing – original draft; data curation; conceptualization. **Huma Dar:** Conceptualization; methodology; software. **Tommy Fornander:** Conceptualization; methodology; investigation; supervision; funding acquisition. **Gizeh Perez‐Tenorio:** Methodology. **Helena Fohlin:** Conceptualization; methodology; data curation. **Olle Stål:** Conceptualization; methodology; supervision; funding acquisition; writing – review and editing. **Julia Tutzauer:** Writing – review and editing; formal analysis; writing – original draft; validation; methodology; visualization. **Linda S. Lindström:** Conceptualization; methodology; software; data curation; supervision; funding acquisition.

## FUNDING INFORMATION

This work was supported by the Swedish Research Council [Vetenskapsrådet, grant number 2020‐02466 and 2023‐03009 to LSL]; the Gösta Milton Donation Fund [Stiftelsen Gösta Miltons donationsfond, to LSL]; the Swedish Cancer Society [Cancerfonden, grant number 222081, 220552SIA to LSL]; Stockholm Cancer Society [Cancerföreningen i Stockholm, grant number 181093 to TF, 221233 and 201212 to LSL]; The Swedish Cancer Society [Cancerfonden grant No. 222216 to OS]; Lions Cancer Fund West[Lions Cancerfond West grant number 2023:8 to AN]; Swedish Breast Cancer Foundation [Svenska Bröstcancerförbundet to AN]; and the King Gustav the Vth Jubilee Clinic Cancer Foundation in Gothenburg [Jubileumsklinikens Cancerfond grant number 2022:414 to AN].

## CONFLICT OF INTEREST STATEMENT

The authors declare no conflicts of interest.

## ETHICS STATEMENT

The research was performed in accordance with the Declaration of Helsinki. Informed consent was obtained before random assignment, and the STO‐3 trial was approved by the Karolinska Institutet regional ethics committee with the Stockholm Regional Cancer Center as the trial center.

## Supporting information


**Data S1.** Supporting Information.

## Data Availability

Restrictions apply to the availability of these data according to GDPR. Data were obtained from the STO Trialist Group and are available from the authors with the permission from the STO Trialist Group.
