# Supplementary material for: Long‐term benefit from adjuvant tamoxifen therapy for ER+ HER2− breast cancer by PR positivity
Source: Int J Cancer. 2026 Mar 5;159(2):450–9. doi: 10.1002/ijc.70409 (PMC13193497; doi:10.1002/ijc.70409)
Supplement: Supplementary file 1 — Data S1. Supporting Information. [file IJC-159-450-s001.pdf]

Supplementary Material for

Long-term Benefit from Adjuvant tamoxifen Therapy for ER+ HER2- Breast Cancer by PR positivity

Anna E. Nordenskjöld, Magdalena Ríos-Romero,Huma Dar, Tommy Fornander, Gizeh Perez-Tenorio, Helena Fohlin, Olle Stål, Julia Tutzauer, and Linda S Lindström

Table of contents:

Supplementary figures:

Figure 1: Kaplan-Meier analyses of 25-years DRFI for adjuvant tamoxifen treated respectively not treated patients divided into PR-positive and PR-negative groups

Figure 2:Kaplan-Meier analyses of 25-years DRFI for adjuvant tamoxifen treated respectively not treated patients divided into PR-positive and PR-negative groups according to ASCO guidelines

Figure 3: Kaplan-Meier analyses of 25-years DRFI for patients with Luminal A tumors of adjuvant tamoxifen treated respectively not treated patients divided into PR-positive and PR-negative groups

Supplementary table:

Multivariable Cox proportional analyses of 25-years DRFI for adjuvant tamoxifen treated respectively not treated patients divided into PR-positive and PR-negative groups according to ASCO guidelines

SUPPLEMENTARY FIGURES

Supplementary Figure 1. Kaplan-Meier analyses of 25-years Distant Recurrence-Free Interval (DRFI) in ER-positive HER2-negative patients randomly assigned to at least 2 years of tamoxifen therapy compared with no adjuvant endocrine therapy (control). Pairwise comparison of tamoxifen therapy benefit for the PR-based subgroups. (A) PR-positive status by IHC; (B) PR-negative status by IHC; (C) PR high H Score; (D) PR low H Score; (E) PR high gene expression; and (F) PR low gene expression.

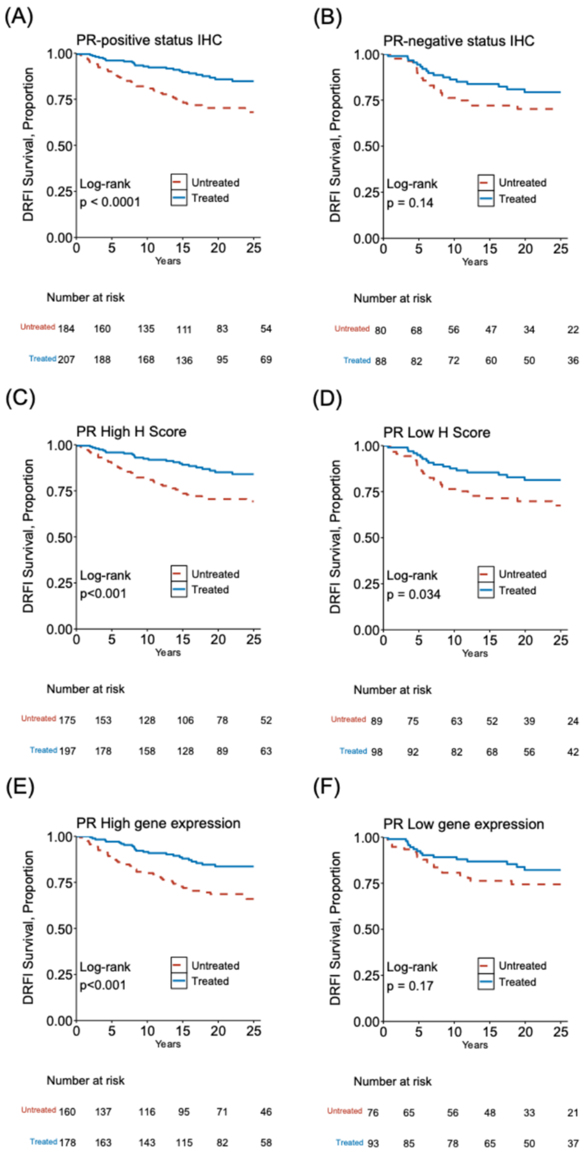

Supplementary Figure 2. Kaplan-Meier analyses of 25-years Distant Recurrence-Free Interval (DRFI) in ER-positive HER2-negative patients randomly assigned to at least 2 years of tamoxifen therapy compared with no adjuvant endocrine therapy (control). (A) PR status by IHC was reclassified according to the current ASCO guidelines. Pairwise comparison of (B) PR-positive and (C) PR-negative patients by IHC according to the current ASCO guidelines. Luminal A patients were not included due to limited sample sizes.

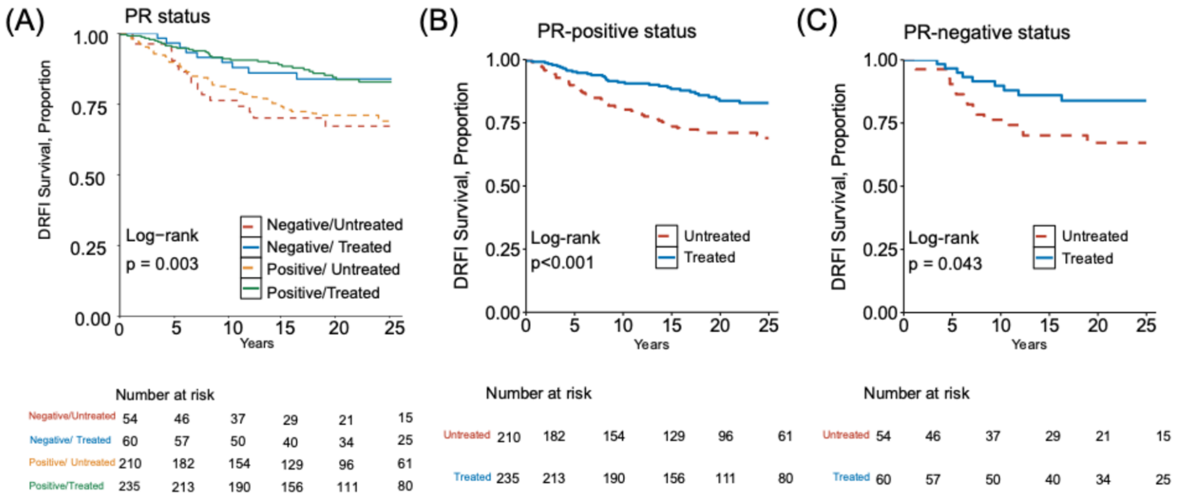

Supplementary Figure 3. Kaplan-Meier analyses of 25-years Distant Recurrence-Free Interval (DRFI) in Luminal A patients randomly assigned to at least 2 years of tamoxifen therapy compared with no adjuvant endocrine therapy (control). Pairwise comparison of tamoxifen therapy benefit for the PR-based subgroups. (A) PR-positive status by IHC; (B) PR-negative status by IHC; (C) PR high H Score; (D) PR low H Score; (E) PR high gene expression; and (F) PR low gene expression.

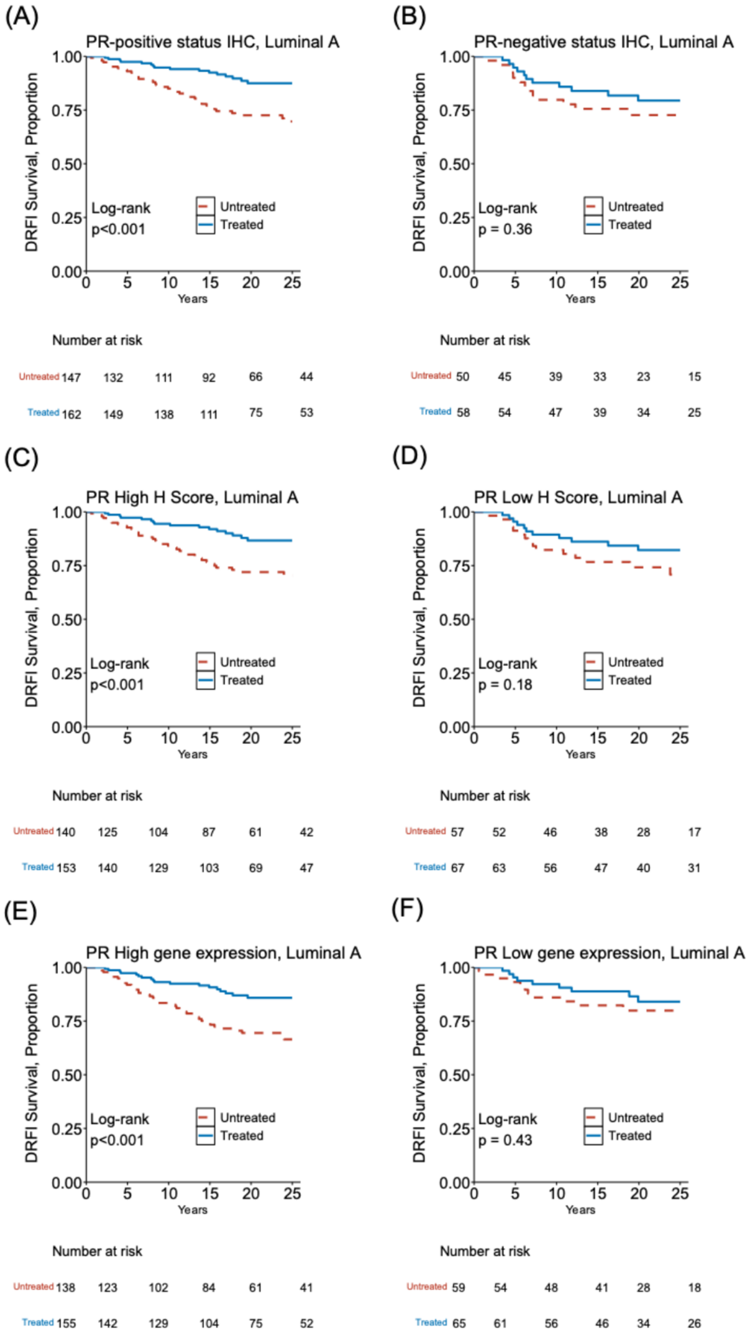

Supplementary Table 1. Multivariable Cox proportional hazard regression analysis of 25-year distant recurrence-free interval (DRFI) contrasting patients randomized to tamoxifen versus control. PR status by IHC was reclassified according to the current ASCO guidelines.

All ER-positive/HER2-negative patients are presented. Luminal A patients were not included due to limited sample sizes. Bold and asterisks indicate significant P<0.05.

	Trial arm	Patients No.	Distant recurrences 30 years No.	Risk of Distant recurrence HR (95% CI)[a]
ER+ HER2- patients				
PR pos	Tamoxifen	216	31	**0.45 (0.29-0.71)***
	Control	201	55	1.00 (ref.)
PR neg	Tamoxifen	54	9	**0.38 (0.15-0.94)***
	Control	49	15	1.00 (ref.)

[a] Adjusted for age, randomization period, tumor size, tumor grade, Ki-67 status and type of surgery. DR=distant recurrences; aHR=adjusted hazard ratio.
